# Long-lasting anxiolytic effect of neural precursor cells freshly prepared but not neurosphere-derived cell transplantation in newborn rats

**DOI:** 10.1186/1471-2202-15-94

**Published:** 2014-08-02

**Authors:** Simone Amaro Alves Romariz, Daisyléa de Souza Paiva, Maria Fernanda Valente, Gabriela Filoso Barnabé, Roberto Frussa-Filho, Regina Cláudia Barbosa-Silva, Maria Elisa Calcagnotto, Beatriz Monteiro Longo

**Affiliations:** Departamento de Fisiologia, UNIFESP, Rua Botucatu, 862, 5° andar, 04023-062 São Paulo, SP Brazil; Departamento de Farmacologia, UNIFESP, Rua Botucatu, 862, 04023-062 São Paulo, SP Brazil; Departamento de Biociências, UNIFESP, Rua Silva Jardim, 136, 11015-020 Santos, SP Brazil; Departamento de Bioquímica, Instituto de Ciências Básicas da Saúde, UFRGS, Rua Ramiro Barcelos 2600, 90035-003 Porto Alegre, RS Brazil

**Keywords:** Anxiety, Medial ganglionic eminence, Neuronal precursor cells, Transplantation

## Abstract

**Background:**

The GABAergic system plays an important role in modulating levels of anxiety. When transplanted into the brain, precursor cells from the medial ganglionic eminence (MGE) have the ability to differentiate into GABAergic interneurons and modify the inhibitory tone in the host brain. Currently, two methods have been reported for obtaining MGE precursor cells for transplantation: fresh and neurosphere dissociated cells. Here, we investigated the effects generated by transplantation of the two types of cell preparations on anxiety behavior in rats.

**Results:**

We transplanted freshly dissociated or neurosphere dissociated cells into the neonate brain of male rats on postnatal (PN) day 2–3. At early adulthood (PN 62–63), transplanted animals were tested in the Elevated Plus Maze (EPM). To verify the differentiation and migration pattern of the transplanted cells *in vitro* and *in vivo*, we performed immunohistochemistry for GFP and several interneuron-specific markers: neuropeptide Y (NPY), parvalbumin (PV) and calretinin (CR). Cells from both types of preparations expressed these interneuronal markers. However, an anxiolytic effect on behavior in the EPM was observed in animals that received the MGE-derived freshly dissociated cells but not in those that received the neurosphere dissociated cells.

**Conclusion:**

Our results suggest a long-lasting anxiolytic effect of transplanted freshly dissociated cells that reinforces the inhibitory function of the GABAergic neuronal circuitry in the hippocampus related to anxiety-like behavior in rats.

**Electronic supplementary material:**

The online version of this article (doi:10.1186/1471-2202-15-94) contains supplementary material, which is available to authorized users.

## Background

Neural stem cell-based therapies have recently been proposed as a possible therapeutic strategy targeted to modulate hyperexcitability by increasing inhibitory neuronal activity in the central nervous system [1, 2]. Neural precursor cells from the medial ganglionic eminence (MGE) of the telencephalic region in the developing brain are responsible for producing most of the inhibitory interneurons in both the cortex and the hippocampus of the mature brain [3, 4]. When transplanted into the newborn cortex, these cells are able to migrate and integrate into the circuitry of the host brain, forming functional synapses that modify the inhibitory input [3–5]. In the central nervous system, GABA-mediated inhibition is critical for controlling synaptic circuits involved in the modulation of anxiety [6]. Studies evaluating the anxiety behavior of animals transplanted with cells derived from the MGE [5] and LGE (lateral ganglionic eminence) [7] have shown that these cells can produce anxiolytic-like effects.

Two methods have been reported for obtaining MGE precursor cells before transplantation: freshly dissociated cells [3, 8] and cultured as neurosphere dissociated cells [9]. Neurospheres consist of cell aggregates that are cultivated in suspension in the presence of growth factors, and they are able to differentiate into three cell types of the central nervous system: neurons, astrocytes and oligodendrocytes [10]. *In vitro* studies using MGE cells cultured as neurospheres have shown that these cells are able to maintain their regional identity, giving rise to inhibitory interneurons besides presenting other neural lineages such as astrocytes and oligodentrocytes [11–14]. The fresh dissociation method consists of transplanting the fetal brain cells immediately after dissecting them from the tissue. In this method, the cells migrate widely, maintaining their inhibitory phenotype and functionality [3, 5, 8, 15]. When transplanted into the cerebral cortex, these cells are able to migrate and restore hippocampal function [16, 17].

Because of the increasing number of emerging studies using MGE cells prepared by different methods and showing discrepant results, the aim of the present study was to compare the two preparations of MGE cells and investigate the long-lasting effects of the transplantation of either freshly dissociated or neurosphere dissociated MGE-derived cells into the neonate brain of male rats on postnatal (PN) day 2–3 on the anxiety behavior of these animals when tested at early adulthood (PN 62–63) using the Elevated Plus Maze (EPM). The EPM is one of the most widely used tests in contemporary preclinical research on anxiety, and is based on an innate fear that rodents have for open and elevated spaces [18]. When in the EPM, rats tend to avoid the open arms and clearly prefer the enclosed arms. The avoidance of the open arms occurs primarily because the open arms prevent the rat from engaging in thigmotaxic behavior [19]. The ratio of open-arm to total arm entries has been used as an index of anxiety [20]. Often, the percentage of time spent in the open arms is also reported. Anxiolytic drugs increase the number of entries into and the total time spent in the open arms, whereas anxiogenic agents do the opposite [21, 22]. Our data suggest a long-term anxiolytic effect following transplantation of freshly dissociated MGE cells, but not of cells expanded as neurospheres. We propose that the fresh cells were able to reinforce the inhibitory function of the GABAergic neuronal circuitry related to anxiety-like behavior in rats.

## Methods

All animals were maintained in accordance with the Guide for the Care and Use of Laboratory Animals (National Research Council). All experimental protocols were approved by the Animal Care and Ethics Committee of UNIFESP/SP (CEP 0081/09).

Sprague Dawley transgenic rat embryos (E14.5) expressing enhanced green fluorescent protein (SD-Tg (GFP) 2BalRrrc), obtained from Charles River Labs and provided by CEDEME (Center for the Development of Animal Models in Biology and Medicine at Universidade Federal de São Paulo) were used as the MGE cell donors.

### Fresh dissociation preparation

For tissue extraction and cell dissociation, ventricular and subventricular layers of the MGE were dissected from E14.5 rat embryos expressing enhanced green fluorescent protein (GFP). Briefly, the tissue was removed and mechanically dissociated by pipetting and centrifugation; the cells were washed with DMEM/ F-12 (Dubelco’s Modified Eagle Medium, Gibco) containing DNase I (10 mg/mL), centrifuged, and ressuspended in the same medium. We determined the cell number and viability of live cells using Trypan Blue exclusion method. The cell density was adjusted to ~100,000 cells/μL of viable cells (90% of cell viability) in culture medium per animal. To verify the *in vitro* cell differentiation into GABAergic neurons, part of the cells was plated in laminin/poly-l-lysine to confirm the GABA phenotype (Additional file 1: Figure S1).

### Neurosphere culture preparation

Ventricular and subventricular layers of the MGE were dissected from E14.5 rat embryos expressing enhanced green fluorescent protein (GFP). The tissue was removed and incubated in trypsin for 5 minutes at 37°C followed by inactivation with fetal bovine serum (Gibco). The tissue was dissociated until a cell suspension was obtained. After centrifugation, the pellet was ressuspended in 0.5 mL of DMEM/F-12. To check the number and cell viability, cells were counted in a Neubauer chamber in the presence of trypan blue. The cells were cultured as neurospheres in a density of 100,000 cells/ml. The culture medium consisted of DMEM/ F-12 supplemented with 1% N2 (100× Invitrogen), 1% L-glutamine (200 mM, Invitrogen), EGF (20 ng/ml, Sigma), FGF-2 (10 ng/mL, R & D Systems) and penicillin (100 IU/ml), streptomycin (100 μg/mL) and amphotericin B (0.25 μg/mL) (all in antibiotic-antimycotic solution 100×, Invitrogen). Cells were maintained in culture between 5 and 7 days. To verify the *in vitro* cell differentiation into GABAergic neurons, part of the cells was plated to confirm the GABA phenotype (Additional file 1: Figure S1).

### Neonatal Transplantation

A suspension of freshly dissociated or neurosphere dissociated cells was placed in a Narishige microinjector guided by a stereotaxic apparatus. Male Sprague Dawley rat pups (PN2-3) were anesthetized by hypothermia (−4°C), and 5–10 × 10^4^ GFP^+^ cells in 0.6 μL were bilaterally injected into the cortex of each rat for both methods of cell preparation. The approximate bregma co-ordinates of the cortex targeted for injections were (2.4 mm A, 3.2 mm L, 1.2 mm D) from bregma. Animals from the group named MGE-F received freshly dissociated MGE cells (n = 7), and an age-matched control (CTRL-MF) group received culture medium (DMEM-F12) only (n = 8) in the same site and volume. Animals in the MGE-N group received neurosphere dissociated MGE-derived cells (n = 16), and an additional control group received culture medium (CTRL-MN, n = 14). An age-matched control group (CTRL-MF and CTRL-MN) was established with each experimental group and was transplanted on the same day as the respective experimental groups (MGE-N or MGE-F). To avoid differences in basal levels of stress and anxiety, animals from the control and experimental groups were from the same litter, and all pups remained away from their mothers for the same amount of time.

### Elevated Plus Maze (EPM)

The EPM has been used extensively to evaluate anxiety-related behavior in rodents [23, 24]. It consisted of two open (50 × 10 cm) and two enclosed (50 × 10 × 40 cm) arms made of wood, arranged in such a way that the two pairs of identical arms were opposite to each other. The open arms were bounded by 1 cm-high ledge on the border of the arms. The maze was raised to a height of 50 cm above the floor. Each animal was placed in the center of the apparatus facing a closed arm, and the number of entries (arm entry defined as all four paws into an arm) and the time spent in the open and closed arms were recorded for 5 minutes. The percentage of time spent in the open arms and the number of open arm entries were recorded as indices of anxiety-like behavior [21]. The number of entries into either arm of the apparatus was used as an index of general activity [25]. The maze was thoroughly cleaned after each test with a solution of 20% ethanol and then dried. Each rat was tested only once.

The behavioral tests were performed at 60 days after the transplantation with either MGE-derived cells (fresh or neurosphere dissociated cells) or medium. Tests were performed during the light period (14:00–17:00 h). The behavior of each animal was videotaped by a video camera located 150 cm above the apparatus. The videos were later watched and analyzed by a trained observer who was blind to the experimental condition.

### Immunohistochemistry

The animals were deeply anesthetized and perfused through the heart with 100 mL of phosphate-buffered saline (PBS) followed by 250 mL of 4% paraformaldehyde. Coronal cryostat sections (30 μm thick) were made between bregma 0.98 and bregma −3.28 mm, according to the stereotaxic coordinates of the rat brain atlas [26]. The precise bregma co-ordinates of the analyzed coronal sections for the cortex and hippocampus were between bregma −2.28 and −5.40 [26]. From this interval, we selected 5 sections for each structure, that would be equivalent to the dorsal, medial and ventral hippocampus (−2.36, −3.32, −4.00, −4.74 -5.38) of this interval of bregma coordinate. To identify MGE-derived inhibitory neurons in the transplanted brains, the sections were incubated with interneuronal markers for neuropeptide Y (NPY), calretinin (CR) and parvalbumin (PV). The sections were incubated for 2 h with anti-GFP AlexaFluor 488-conjugated antibody (1:600; Molecular Probes/Invitrogen), followed by an overnight incubation with rabbit antibodies against NPY (1:2000, Molecular Probes), CR (1:2000, Molecular Probes) and PV (1:2000, Molecular Probes). The sections were then incubated with anti-rabbit IgG AlexaFluor 546-conjugated antibodies (1:600, Molecular Probes/Invitrogen) for 1 hour. The sections were mounted using a nuclear-counterstaining, fluorescence-preserving mounting medium containing DAPI (Vector Labs). The slides were examined using fluorescence (Nikon 80i) and confocal (Olympus FluoView 1000) microscopes. For each section, double-labeled cells were qualitatively evaluated for their localization in the brain areas of interest (the cortex and hippocampus) and for their morphology.

In order to quantify the MGE-derived transplanted cells, the sections were incubated with anti-GFP AlexaFluor 488-conjugated antibody (1:600; Molecular Probes/Invitrogen) and were mounted on slides and sealed with coverslips. The sections were examined using a fluorescence microscope (Nikon 80i), and images were captured and digitized using the Nikon ACT-1 v.2 system. The estimation of GFP^+^ cells was sampled in 5 coronal sections per animal for cortex and hippocampus. Images of 10 random, non-overlapping fields were captured and quantified for each region under 20× magnification.

### Statistical analysis

Statistical analyses of anxiety-related behavior and cell counts were performed using the Mann–Whitney test (SPSS XI.) for comparison between the MGE-N x CTRL-MN and MGE-F x CTRL-MF groups. A significance level of 5% was assumed. Data are shown as the mean ± SE.

## Results

### Behavioral results

**Figure 1:**
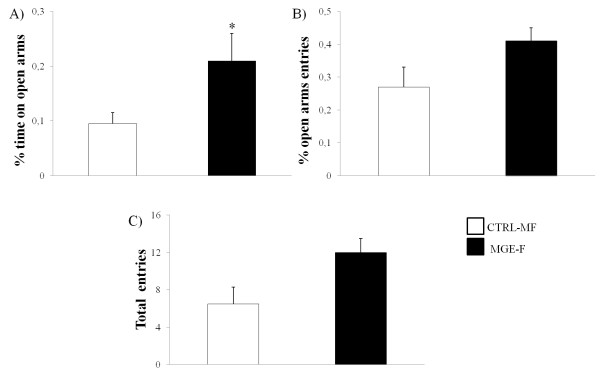
**Elevated plus maze (EPM) using animals transplanted with freshly dissociated cells.** EPM data comparing MGE-F (n = 7) and CTRL-MF (n = 8) groups; **A)** Percentage of time spent in the open arms; **B)** Percentage of entries into the open arms; **C)** Total number of entries into the open and closed arms. Data are shown as the mean and standard error. (*) Significant difference between groups (p < 0.05), Mann–Whitney test.

**Figure 2:**
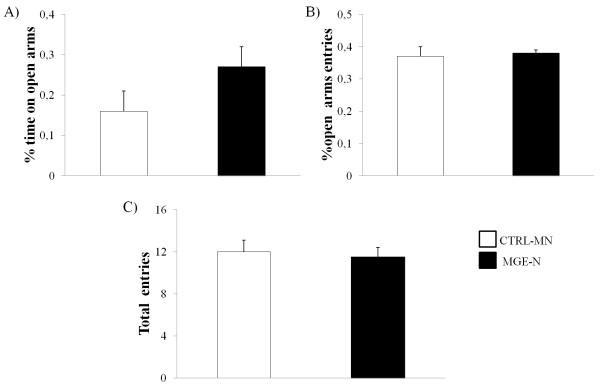
**Elevated plus maze (EPM) using animals transplanted with neurosphere cells.** EPM data comparing MGE-N (n = 14) and CTRL-MN (n = 16) groups; **A)** Percentage of time spent in the open arms; **B)** Percentage of entries into the open arms; **C)** Total number of entries into the open and closed arms. Data are shown as the mean and standard error, Mann–Whitney test.

In order to assess the inhibitory effects of transplanted cells *in vivo*, the anxiety-like behavior of the animals was evaluated using the EPM 60 days after transplantation and compared to an age-matched control group. Rats from the MGE-F group showed a significant increase in the percentage of time spent in the open arms (p = 0.04), indicating an anxiolytic effect as compared to the CTRL-MF group (Figure 1A). The percentage of open arm entries was similar for all groups but with a trend towards an increase in the number of open arm entries in the MGE-F group (p = 0.08) (Figure 1B).The percentage of open arm entries (p = 0.9) and the total time spent in the open arms (p = 0.4) for animals that received neurosphere cells (MGE-N) were not different from the control group (CTRL-MN), indicating that MGE cells grown in culture were not able to generate an anxiolytic effect (Figure 2A and 2B).

Concerning the total number of entries into both the open and closed arms, no significant differences were found between the groups MGE-F *versus* CTRL-MF (p = 0.2) (Figure 1C) and MGE-N *versus* CTRL-MN (p = 0.7) (Figure 2C). These data suggest that there was no alteration in general locomotor activity and that the preference for the open arms was related to the anxiolytic effect of the freshly prepared MGE cells.

### Analysis of transplanted GFP^+^ cells

**Figure 3:**
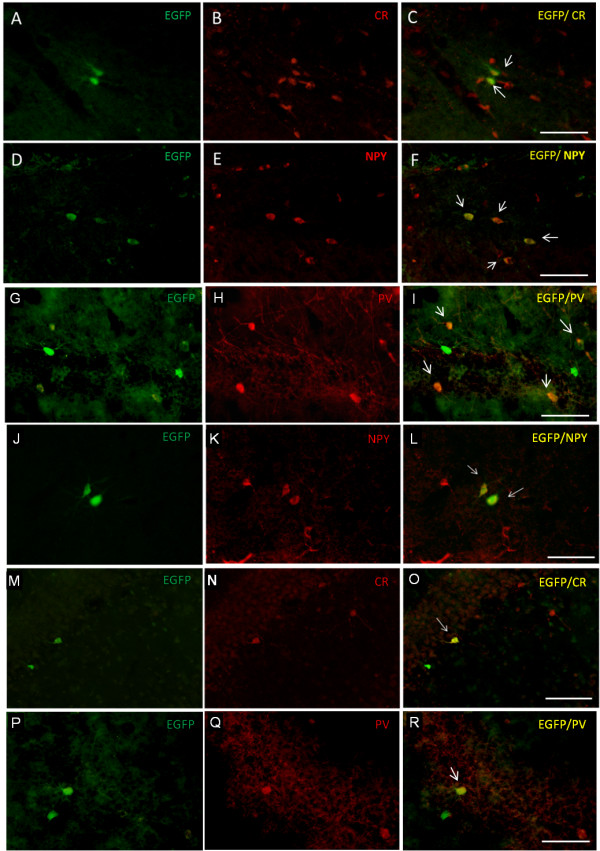
**Double-labeled cells with GFP and inhibitory interneuronal markers.** Confocal images revealed double labeling of GFP^+^ cells and inhibitory interneuronal markers in animals that received freshly dissociated cells **(A-I)** and neurosphere cells **(J-R)**. The interneuronal markers used were: calretinin (CR) **(A-C)** and neuropeptide Y (NPY) in the hilus **(D-F)**; parvalbumin (PV) in the hilus **(G-I)**; neuropeptide Y (NPY) in the cortex **(J-L)**; calretinin (CR) **(M-O)** and parvalbumin (PV) **(P-R)** in the CA1. Scale bar = 50 μm.

A qualitative evaluation of the MGE-transplanted cells in the host brain was performed by immunohistochemistry to confirm the presence of GFP^+^ transplanted cells, as well as to verify their inhibitory phenotype by co-localization with the interneuronal markers NPY, PV and CR. The analysis of GFP^+^ cells in the adult brain indicated that the MGE-derived cells from both the freshly dissociated and neurosphere preparations were present and differentiated into inhibitory neurons (Figure 3). Additionally, these cells migrated throughout the parenchyma from the site of the injection (cortical layers 3 and 4) and were mainly found in the cortex and hippocampus, with an insignificant number of GFP^+^ cells detected in other brain regions. The double-labeled NPY^+^/GFP^+^, PV^+^/GFP^+^ and CR^+^/GFP^+^ cells were detected in both the cortex and hippocampus (Figure 3), confirming that the transplanted cells from both preparation types, fresh and expanded as neurosphere, were able to differentiate into inhibitory interneurons *in vivo*.

**Figure 4:**
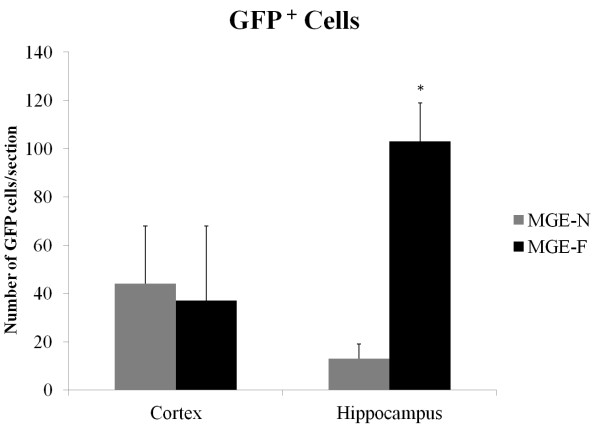
**Quantification of GFP** ^**+**^ **cells.** GFP^+^ cells were quantified for the MGE-F and MGE-N groups in two brain regions: the cortex; and the hippocampus. A significant increase in the number of GFP^+^ cells was detected in the hippocampus of group MGE-F compared to MGE-N. (*) Significant difference between groups (p < 0.05), data are presented as the mean and standard error, Mann–Whitney test.

A quantitative analysis of the amount of GFP^+^ cells within the two groups (MGE-F and MGE-N) showed no significant difference in cortical regions (p = 0.9). In the hippocampus, however, the MGE-F group had a higher number of GFP^+^ cells when compared to the MGE-N group (p = 0.009). This finding provides a possible explanation for the behavioral results, associating the presence of MGE-F-derived inhibitory neurons in the hippocampus with a reduced anxiety-like behavior (Figure 4).

## Discussion

Our results indicate that MGE-derived cells obtained by two different methods, freshly dissociated or neurosphere dissociated, and then transplanted into the neonate brain produce distinct anxiety-related behaviors in adult rats. Animals from the MGE-F group presented an anxiolytic-like behavior, whereas transplanted neurosphere-derived MGE cells was not able to decrease the basal levels of anxiety. The freshly dissociated cells transplanted into newborn rats showed a long-lasting effect on anxiety behavior when evaluated two months later in adulthood. This effect could be attributed to the differentiation of these cells into inhibitory GABAergic neurons that were able to migrate mainly to the hippocampus, while the cells from the neurospheres remained in the cortex with no sign of long distance migration.

The GABAergic system plays an important role in modulating the levels of anxiety. Investigations using animal models are currently being conducted to better understand the GABAergic circuits involved in the regulation of anxiety levels and mood disorders. Moreover, cells obtained from MGE could present discrepant results when transplanted as fresh or neurosphere cells. Recent studies have shown that cells derived from the sub regions of eminences were able to modify the anxiety behavior in transplanted animals increasing the GABAergic transmission and producing anxiolytic effects [5, 7]. In the present study, although animals transplanted with neurosphere cells did not present effects in anxiety-like behavior, animals grafted with MGE fresh cells presented a clear-cut anxiolytic-like behavior in the EPM test. The anxiolytic effect was observed 60 days after transplantation, sufficient time for cells migrate, acquire an inhibitory phenotype and to integrate into host tissue [3]. These long-term effects of fresh cell transplantation in anxiety-like behavior are consistent with our previous study with fresh MGE cells [5] that described the same decrease in anxiety levels in two different ages, and showed a high density of transplanted cells mainly in the striatum, corpus callosum and hippocampus.

The presence of a large number of inhibitory neurons in the hippocampus obtained from the fresh dissociation method two months after transplantation likely contributes to decreased levels of anxiety. The hippocampus is a structure of extreme importance, not only for learning and memory but also for anxiety and fear-related responses, being involved either directly or indirectly in the modulation of anxiety [27–29]. The hippocampus is linked to a number of subcortical structures including the amygdala, and it has been associated with certain aspects of emotional behavior, anxiety in particular [30]. Moreover, it has been reported that animals with impaired hippocampal neurogenesis have increased levels of anxiety, showing a direct correlation between hippocampal plasticity and the modulation of anxiety [31].

The GFP^+^ cells obtained from the neurosphere preparation also differentiated into GABAergic inhibitory interneurons, as evidenced by the double-labeling with markers for NPY, PV and CR along with GFP. Although specific studies have succeeded in obtaining a considerable number of neurons after the transplantation of neurospheres in classic neurogenic areas of the brain [32, 33], few neurons have been found when these cells were transplanted into areas not usually described as neurogenic, such as the striatum [13, 34, 35]. Thus, the environment may greatly influence the differentiation of cells from neurospheres [36]. Furthermore, although grafts of neurosphere cells derived from the MGE preserved their ability to generate inhibitory interneurons *in vitro* and after transplantation, they primarily differentiate into astrocytes [13, 14]. In our hands, even though the neurosphere-derived cells were alive and presented an inhibitory phenotype, they were not able to reduce the basal levels of anxiety. Indeed, these cells failed to migrate to the hippocampus, a region involved in anxiety behavior, and instead remained in the cortex, which was the site of transplantation.

One could suggest that the low number of MGE cells from neurosphere found in the two areas can be explained by the cell death that must occur between the transplantation in postnatal period and the end of the experiment 60 days after. Although the number of injected cells was the same for both preparations, e differences in anxiety behavior could be caused by differences in cell death rate between the two cells after transplantation, and that the anxiolytic effect can lie in the reduced apoptosis rate of the fresh cells, However, we have to consider that the apoptosis would have occurred soon after the transplantation (for the vast majority of cells), and could not be detected long after the transplantation of cells, when the anxiety test was applied (60 days after).

The changes in the anxiety levels of animals that were transplanted with freshly dissociated MGE cells indicate that these cells were able to not only differentiate into inhibitory neurons but also integrate into the local circuitry and change the inhibitory tone 60 days after the transplantation. By contrast, the transplanted cells dissociated from neurospheres were able to differentiate into inhibitory interneurons but were unable to induce changes in the levels of anxiety, probably because the amount of cells that differentiated into inhibitory interneurons specifically in the hippocampus was insufficient to generate an anxiolytic effect. According to our data, even though the neurosphere-derived cells were alive and presented inhibitory neuron phenotype, they have failed to migrate to the hippocampus, what can explain their incapability to modulate anxiety.

## Conclusion

Our results demonstrate a long-term anxiolytic effect induced by the transplantation of freshly dissociated MGE-derived cells but not by neurosphere dissociated cells. The freshly dissociated MGE cells were able to modulate the inhibitory tone in the hippocampus, as evidenced by a long-lasting decrease in the levels of anxiety of transplanted animals. This work provides a new neural stem cell approach for the study of mood disorders.

## Electronic supplementary material

Additional file 1: Figure S1: *In vitro* cells double-stained for GABA and DAPI. Images from fluorescence microscopy reveled GABAergic interneurons. In (A-C) cells dissociated from neurospheres; and in (D-F) fresh-dissociated cells. Scale bar = 100μm. (TIFF 852 KB)
