# Supplementary figures and images for: Long-lasting anxiolytic effect of neural precursor cells freshly prepared but not neurosphere-derived cell transplantation in newborn rats
Source: BMC Neurosci. 2014 Aug 2;15:94. doi: 10.1186/1471-2202-15-94 (PMC4131043; doi:10.1186/1471-2202-15-94)

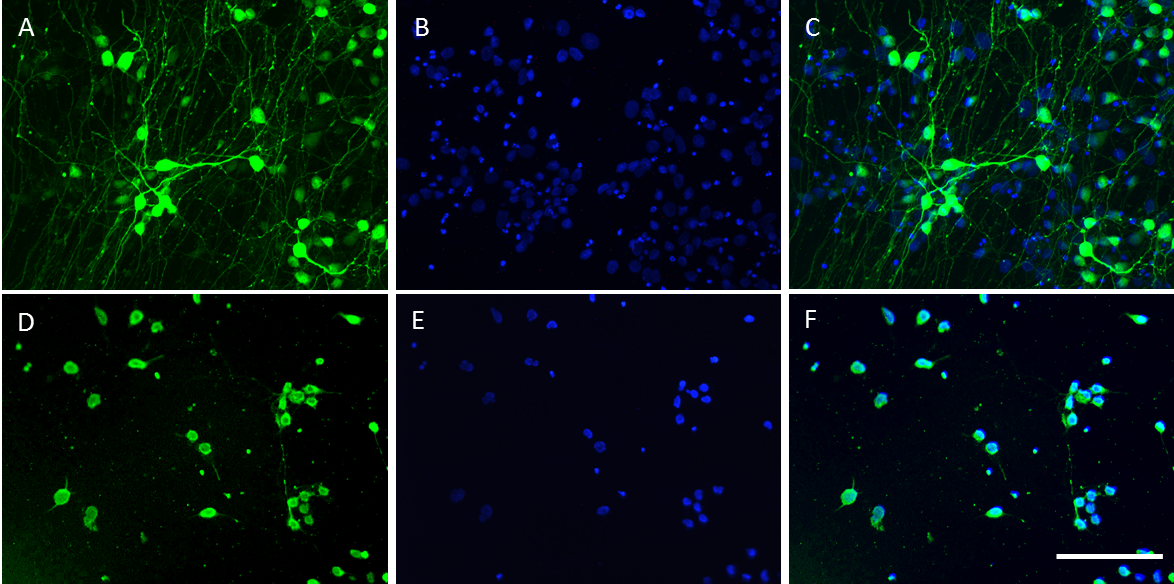

Supplement: Supplementary file 1 — Additional file 1: Figure S1: In vitro cells double-stained for GABA and DAPI. Images from fluorescence microscopy reveled GABAergic interneurons. In (A-C) cells dissociated from neurospheres; and in (D-F) fresh-dissociated cells. Scale bar = 100μm. (TIFF 852 KB) [file 12868_2014_3782_MOESM1_ESM.tiff]
